# Intradermal Glycine Detection with a Wearable Microneedle Biosensor: The First In Vivo Assay

**DOI:** 10.1021/acs.analchem.2c02317

**Published:** 2022-08-18

**Authors:** Qianyu Wang, Agueda Molinero-Fernandez, Ana Casanova, Joep Titulaer, Jonatan C. Campillo-Brocal, Åsa Konradsson-Geuken, Gaston A. Crespo, Maria Cuartero

**Affiliations:** † Department of Chemistry, School of Engineering Sciences in Chemistry, Biotechnology and Health, KTH Royal Institute of Technology, Teknikringen 30, SE-100 44 Stockholm, Sweden; ‡ Section of Neuropharmacology and Addiction Research, Department of Pharmaceutical Biosciences, Uppsala University, SE-751 05 Uppsala, Sweden; § Department of Genetics and Microbiology, University of Murcia, Campus Universitario de Espinardo, 30100 Murcia, Spain

## Abstract

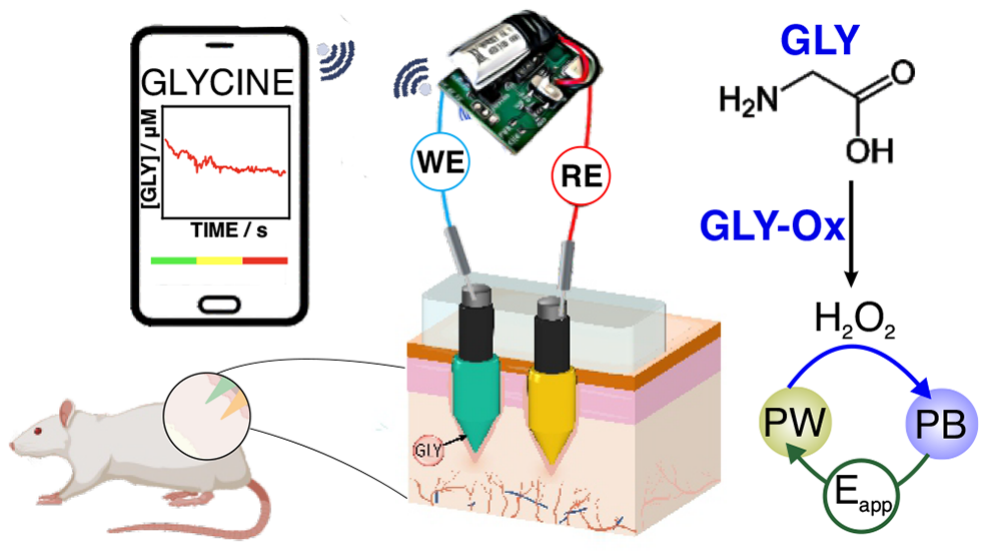

Glycine (GLY) is gaining importance in medical diagnoses due to its relationship with multiple physiological functions. Today, GLY is exclusively analyzed using instrumentation centralized in clinical labs, and a tangible point-of-care tool that gathers real-time data from the patient for effective and fast evaluations is lacking. Relevant clinical advances are expected as soon as the rapid provision of both punctual and continuous measurements is possible. In that context, this work presents a microneedle (MN)-based biosensor for intradermal GLY detection in interstitial fluid (ISF). The MN tip is externally tailored to detect GLY levels through the hydrogen peroxide formed in its reaction with a quinoprotein-based GLY oxidase enzyme. The analytical performance of the MN biosensor indicates a fast response time (<7 s); acceptable reversibility, reproducibility, and stability; as well as a wide linear range of response (25–600 μM) that covers the physiological levels of GLY in ISF. The MN biosensor conveniently exhibits high selectivity for GLY over other compounds commonly found in ISF, and the response is not influenced by temperature, pH, or skin insertions. Validated intradermal measurements of GLY were obtained at the in vitro (with pieces of rat skin), ex vivo (on-body tests of euthanized rats) and in vivo (on-body tests of anesthetized rats) levels, demonstrating its ability to produce accurate physiological data. The developed GLY MN biosensor is skin-wearable and provides reliable, real-time intradermal GLY measurements in ISF by means of a minimally invasive approach.

Point-of-care (POC) testing has become an important concept in healthcare in terms of enabling diagnostics and quick feedback of many clinical cases, featuring ease of function near the patient or even self-examination. POC testing not only relieves congestion in emergency rooms and delays in data provision but also paves the way toward more sustainable medical management.^1^ Accordingly, many POC devices are already available to provide information about an individual’s health. Most of the parameters currently being monitored are physical in nature, such as temperature, breathing frequency, blood pressure, pulse, and heart rate.^2^ Despite the valuable information that (bio)chemical parameters can provide, they are more difficult to be conceived at the POC level and hence are still underrepresented in the field.

One of the most successful examples of a POC device is the glucometer, which reflects the excellence that is sought in POC tools: almost no restrictions on patient use anywhere, while offering total reliability at minimal cost. Yet, the glucometer’s configuration and benefits are continuously evolving, with research and innovation heading primarily in four directions: (i) reducing calibration requirements, (ii) data collection for personalized telemedicine assistance, (iii) automating measurements for continuous monitoring, and (iv) avoiding blood collection (finger prick).^3^ Within this context, microneedle (MN)-based (bio)sensor technology is rapidly evolving into wearable POC tools for both punctual and continuous intradermal measurements. The core hypothesis for the clinical use of MN sensors is that the dermal interstitial fluid (ISF) provides the exact same health-related information as blood. Wearable MN intradermal sensors are in a privileged position of being able to combine valuable clinical information with painless ISF access and thus enabling real-time digitalization, which contrasts sharply with traditional blood tests.

While initial efforts were focused on ISF extraction with hollow structures connected to external sensors and chips (hence, the analytical measurements occurred off-skin), recent research has considered the implementation of electrochemical (bio)sensing concepts directly in the MN tip (external or internal modification).^3^ Seemingly, ISF extraction is not a straightforward task, in addition to the fact that the collected volume is very low (ca. 1 μL) to provide reliable measurements.^4^ Tremendous advances have been achieved in both the design and targets of MN (bio)sensors, with glucose sensors at the forefront of the list.^5,6^ Yet, there are several challenges to be addressed, including new analytes, proper calibration, reliable validation of on-body data, and the managing of ethical permits, which results in only few investigations reaching true in vivo measurements.^7−9^

Amino acids (AAs) are potential targets of MN biosensors, as the clinical interest in their detection has increased in recent years owing to new discoveris.^10^ Indeed, fruitful insights are expected along with the development of analytical tools (e.g., MN biosensors) able to provide discrete and continuous body measurements. Glycine (GLY), the smallest AA, is of particular relevance because it accounts for ca. 12% of the total AAs and 20% of AA nitrogen in body proteins, resulting in its significant association with many diseases and physiological states.^11^ While the potential and benefits of GLY are yet under study, some investigations have been dedicated to the correlations between GLY levels and certain body states, e.g., low blood concentrations of GLY are correlated with obesity, diabetes, insomnia, gout, and schizophrenia,^12^ whereas high concentrations of GLY are associated with nonketotic hyperglycinemia (also known as glycine encephalopathy) and cancer progression.^13,14^

Today, the determination of GLY (mainly in blood) is carried out through laboratory-based techniques, including liquid chromatography, mass spectroscopy, and fluorescence assay kits.^15,16^ While these approaches provide reliable results, the main drawbacks are delayed results and rather significant startup overhead, maintenance, and personnel costs, together with the difficulty of obtaining results in the ISF. Hence, the clinical field would benefit from a wearable MN device that provides on-body, real-time (continuous and/or discrete) GLY measurements, something that, to the best of our knowledge, is lacking in the market and scientific literature.

Herein, we present a solid MN biosensor for the intradermal analysis of GLY in ISF by means of an epidermal patch. The MN tip is externally tailored via a multilayering approach to provide indirect GLY analysis through the detection of hydrogen peroxide (H_2_O_2_) formed in its enzymatic reaction with the glycine oxidize (GLY-Ox) enzyme (a quinoprotein).^17^ Validated intradermal measurements of GLY in ISF are accomplished through the following scenarios: (i) in vitro measurements involving rat skin pieces conditioned in solutions with known GLY concentrations, (ii) ex vivo on-body measurements involving euthanized rats, and (iii) in vivo on-body measurements involving anesthetized rats. Notably, the developed experimental path to investigate and validate the GLY MN biosensor may be conveniently followed by other new MN (bio)sensors toward reliable analytical characterization, which is a common challenge.^3,9^ Then, the presented GLY MN biosensor has enormous potential in terms of providing new clinical advances in the prevention and monitoring of illnesses in the context of contemporary medication therapy. It will also accelerate medical research involving GLY, such as restricted cancer treatment, requiring either discrete or dynamic assessment of GLY in real-time.

## Experimental Section

**Figure 1:**
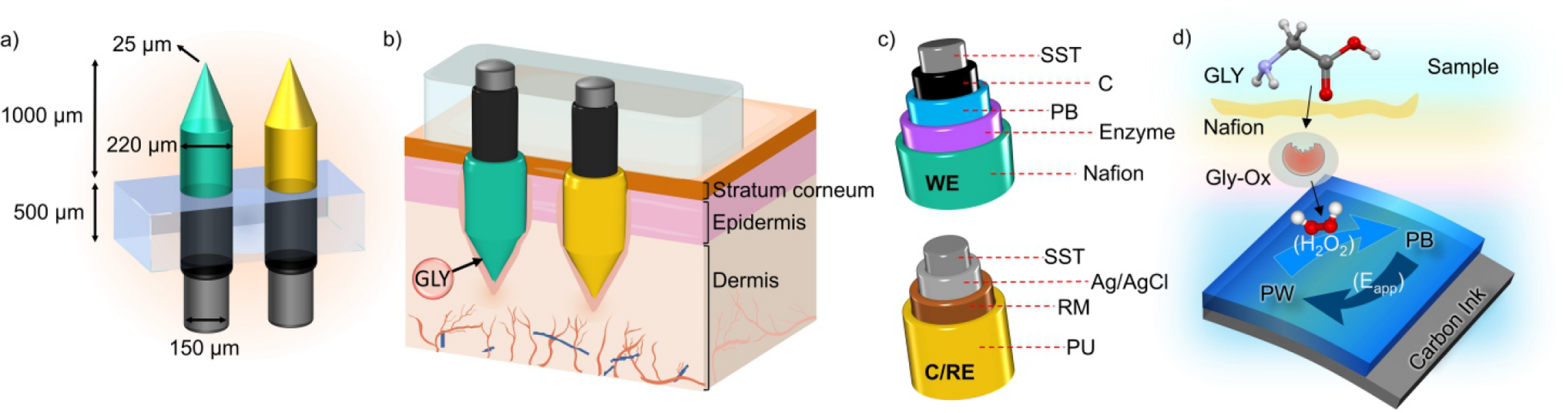
(a) Illustration of the MN patch. (b) Image of the MN patch inserted into the skin. (c) Different layers of the WE and the C/RE. (d) Working GLY biosensor mechanism. GLY = glycine, SST = solid stainless steel, C = carbon, PB=Prussian Blue, RM = reference membrane, PU = polyurethane, PW = Prussian White.

GLY measurements in ISF were accomplished by means of a silicon rubber substrate (Ecoflex 00-50 platinum cure, USA) with two modified MNs (the GLY MN biosensor patch, Figure 1a,b): a working electrode (WE) and a counter/reference electrode (C/RE). To address the issue of skin deformation and viscoelasticity during MN insertion for the in vitro, ex vivo, and in vivo tests, we selected a soft substrate with exceptional flexibility for on-body testing. Commercial stainless-steel alloy MNs were chosen to develop the GLY biosensor due to their simplicity, high mechanical strengths, and low cost.^3^ The total length of the modified MNs (1000 μm) enabled them to penetrate the stratum corneum layer of the skin, thus reaching the dermis ISF and avoiding the blood vessels and nerve endings located at around a depth of 2000 μm (Figure 1b).^18^ The substrate was 13 mm in diameter and 0.5 mm thick. The unmodified stainless-steel solid MNs (Dermaroller, Sweden) were 1.5 mm in length and 150 μM in diameter.

First, the MNs were dip-coated with the corresponding ink (C for the WE, and Ag/AgCl for the C/RE), then cured in an oven (120 °C, 10 min). The MNs were subsequently inserted in the substrate and glued with Loctite Super Glue (Henkel Norden AB) in the upper part (last 0.5 mm in length). From this upper part is where the required electrical connections are further created to the reader. After drying the glue for at least 4 h at room temperature, functionalization was completed in the bottom parts of the MNs. The resulting multilayered MNs are illustrated in Figure 1c.

For the WE, a Prussian Blue (PB) layer was electrodeposited on the C-MN surface by 10 cyclic voltammetry cycles from −0.5 to 0.6 V at 50 mV s^–1^ in a solution comprising 2.5 mM FeCl_3_, 2.5 mM K_3_[Fe(CN)_6_], 100 mM KCl, and 100 mM HCl. This deposition was followed by a 1-h curing at 100 °C in the oven. Next, a GLY-Ox/chitosan (GLY-Ox/CHI) mixture (1:2 v/v ratio of GLY-Ox:CHI) was prepared by diluting the parental GLY-Ox solution (0.97 ± 0.01 U GLY-Ox mL^–1^) three times in phosphate buffer and combining it with CHI (1%, aq.) in 7.15% acetic acid, then drop cast onto the PB-C-MN. This enzyme layer was allowed to dry in the fume hood for at least 1 h. Afterward, 2 μL of 1% wt. Nafion solution (0.2 mL of 5% wt. Nafion in 0.8 mL of water) was drop-casted and allowed to dry for 30 min. Finally, the modified MN was stored in a phosphate buffered saline solution (PBS) at 4 °C, also for between-day measurements.

The C/RE was prepared as reported elsewhere.^19^ Briefly, 3 μL of poly (vinyl butyral) (PVB)/NaCl /MeOH solution (78 mg of PVB, 50 mg of NaCl in 1 mL of methanol) was drop cast on top of the Ag/AgCl film and conditioned in 3 M KCl overnight. Afterward, an external polyurethane (PU) membrane was applied by drop-casting 2 μL of PU solution (20 mg PU in 1 mL THF). All the layers were cured in the fume hood until they were completely dry. The C/RE MN was stored in 3 M KCl.

The bare solid stainless-steel MNs as well as finalized WE and C/RE MNs were characterized using scanning electron microscopy (SEM) images (Figure S1). The dimensions of the initial MNs in terms of length plus widths at the base and tip were confirmed (1.5 mm, 150 and 15 μm, respectively). In the case of the WE, the widths at the base and tip of the MN were found to have increased to 200–220 μm and 24–35 μm, respectively, with the addition of the C, PB, GLY-Ox, and Nafion layers. For the C/RE, the widths at the base and the tip increased to 180–206 μm and ca. 30 μm, respectively, with the implementation of the Ag/AgCl, PVB, and PU layers. The homogeneities of the layers, together with the absence of any physical deterioration in the WE and C/RE MNs after skin insertion, were confirmed. When accompanied by a proper insertion angle and force during the penetration process, the dimensions of our modified MNs should permit painless and minimally invasive skin puncture.^20^

## Results and Discussion

The biosensor patch is composed of two MN-based electrodes, namely, the WE and the C/RE, which are necessary for the amperometry readout (two-electrode system). Figure 1d illustrates the working mechanism for GLY detection. The sample’s GLY is partitioned in the Nafion membrane and diffuses until reaching the GLY-Ox enzyme, where it is then converted into glyoxylate, ammonium, and H_2_O_2_ via a stoichiometric reaction. Meanwhile, for the PB, the original film is first reduced to Prussian White (PW) by applying a constant potential of −50 mV to the electrode, i.e., Fe^3+^/Fe^2+^ moieties, then the generated H_2_O_2_ is spontaneously reduced while the PW is oxidized back to PB. The PB is reduced again to PW via the applied potential, which generates a Faradaic current near the electrode surface in dynamic equilibrium. Any change in the GLY concentration in the sample will change the current’s equilibrium value, which is proportional to the related concentration change over a certain range, thus allowing the quantitative determination of the GLY concentration in the sample. The overall concept was recently reported by our group, providing the first demonstration of an electrochemical biosensing approach to measuring the GLY concentration in urine, sweat, and serum, thanks to the specificity of the GLY-Ox, which was successfully encapsulated in the sensor core.^17^ Following, we translate the GLY biosensing concept for the unprecedented transdermal analysis of GLY in ISF.

### Tailoring of the Components of the MN Biosensor for GLY Detection

**Figure 2:**
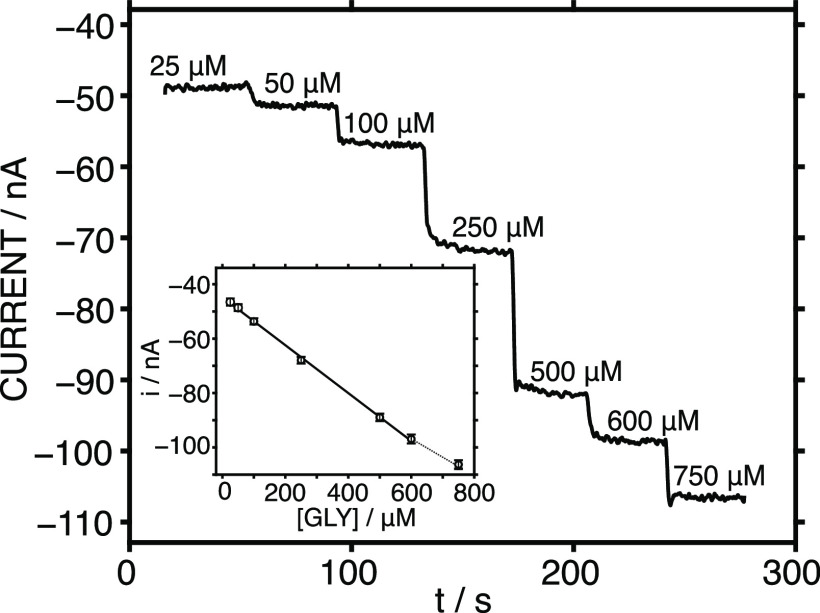
Dynamic amperometric response to increasing H_2_O_2_ concentrations. Inset: average calibration graph observed for three consecutive responses.

Figure 2 displays the amperometric dynamic response of the optimized MN biosensor at increasing GLY concentrations in a PBS background. The response time (*t*_90_) was <7 s across the entire concentration range. The linear range of response (LRR) was from 25 to 600 μM, with a sensitivity (slope) of −0.0880 ± 0.001 nA μM^–1^ (1.5% of variation coefficient within three consecutive calibrations of the same MN biosensor) with an intercept of −51.2 ± 1.1 nA (3.6%). The amperometric response was rather reproducible, even between different MN biosensors (*n* = 7), producing variations of <5.8% for the slope and <10.4% for the intercept (see Figure S2).

After reaching a GLY concentration of 600 μM in solution, the calibration graph begun to deviate from its linearity. There are two possible reasons for this behavior: (i) the enzyme reaches its saturation state, i.e., the limiting factor of the enzymatic reaction rate is the concentration of the enzyme; thus, adding a greater substrate (GLY) concentration does not affect the overall reaction or (ii) the PB layer does not respond to higher H_2_O_2_ concentrations in accordance with its upper limit of detection (upper LOD). The first explanation seems to be the more plausible of the two because the PB-MN electrode presents a wide range of responses toward H_2_O_2_ (see Figure S3) via the PW/PB interconversion. Using a signal-to-noise ratio of 3 in the calibration graph, the LOD was calculated to be 7.9 μM GLY concentration.

The LRR of the MN biosensor included GLY levels within healthy conditions (400–600 μM)^21^ and most of the unhealthy levels,^3,21^ although appropriate clinical data are difficult to locate, likely due to the absence of appropriate analytical tools for ISF measurements. Regarding the expected daily variations, and assuming that blood and ISF GLY present close profiles, fluctuations of more than ±100 μM have been reported in volunteers under different diet conditions.^22^ On the other hand, the administration of a dietary supplementation of GLY has demonstrated to be effective in preventing/treating many disorders, as well as enhancing the sleep quality and neurological operations.^11^ Accordingly, the employment of the developed GLY MN biosensor could be used for achieving personalized therapies via GLY profile monitoring. Both, punctual and continuous GLY measurements are thus valuable toward the replacement of recurrent lab-based daily measurements, additionally pursuing unprecedented clinical data.

Although the achieved LRR seems to be sufficient to assess the just inspected clinical examples, in any specific case where the LRR needs to be expanded toward higher concentrations (such as for cancer-related applications),^23,24^ an additional external polymeric layer could be added to the MN, or the thickness of the Nafion layer could be increased. Using one of these strategies, and acknowledging that a decrease in sensitivity will occur, the number of substrate molecules reaching the enzyme will be restricted, as demonstrated by our group for lactate.^17^ For very low GLY concentrations (<25 μM), the MN biosensor can act as an alarm rather than for quantification.

To obtain the described analytical characteristics, the MN biosensor structure was investigated as follows. First, we evaluated the implementation of a PB layer on the stainless-steel MN to detect H_2_O_2_. We attempted to deposit the PB directly onto the bare MN; however, the formed film was not mechanically stable and was found to be removed during the characterization experiments. This occurred regardless of whether the PB film was electrodeposited or created via drop-casting. We opted for the initial addition of a C layer to the bare MN to facilitate the stronger attachment of the PB film due to the fact that a passive film on the stainless-steel surface (essentially formed by the oxide/hydroxide of chromium and iron) may prevent the direct bonding of the PB.^25^

**Figure 3:**
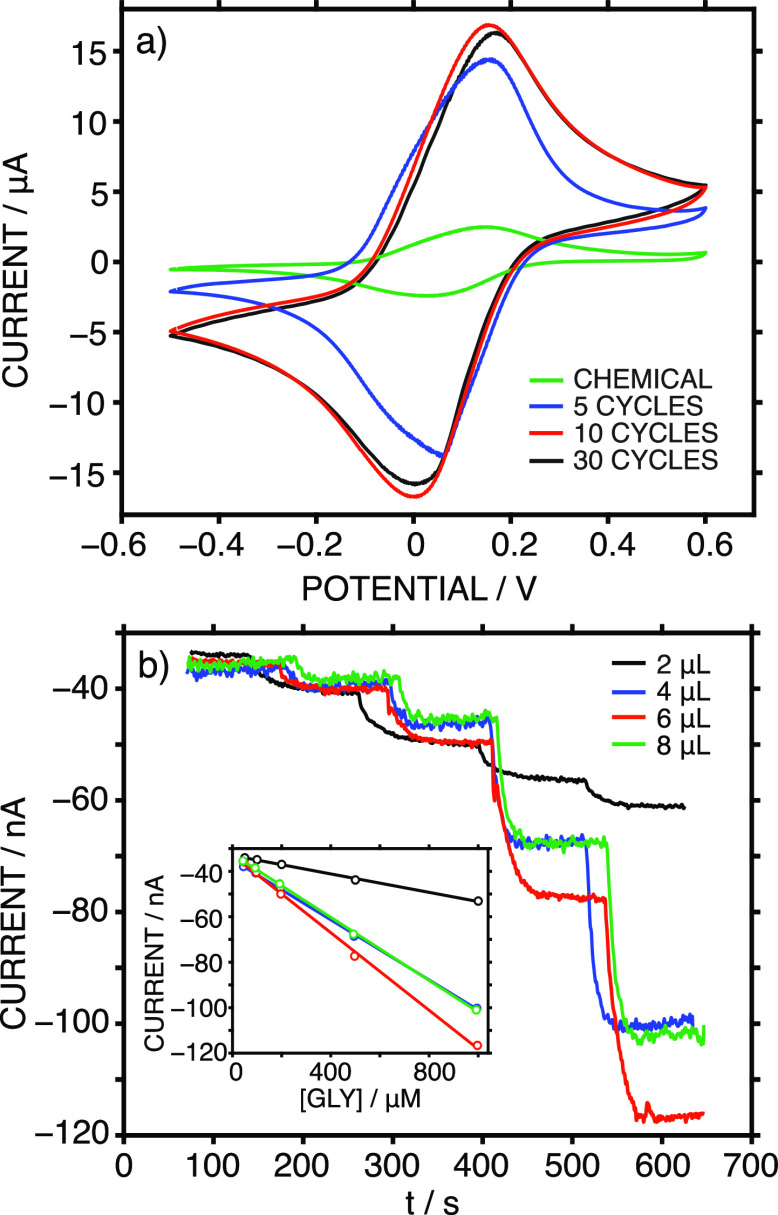
(a) Cyclic voltammograms observed in PBS with differently prepared PB-C-MNs. (b) Amperometric responses observed with Nafion-Enzyme-PB-C-MNs prepared with increasing volumes of the enzyme. Inset: corresponding calibration graphs.

We explored the addition of a PB layer to C-coated MNs via either a chemical reaction or electrodeposition. The chemical reaction was carried out by drop-casting 8 μL of PB precursor solution (0.1 M of FeCl_3_, K_3_[Fe(CN)_6_], KCl, and HCl) onto the MN tip, then allowing 20 min for a reaction at room temperature under dark conditions. Next, the solution was removed, and the MN tip was cleaned in 0.1 M HCl followed by curing for 1 h at 100 °C in an oven. The electrodeposition was performed in a solution containing 2.5 mM FeCl_3_, 2.5 mM K_3_[Fe(CN)_6_], 100 mM KCl, and 100 mM HCl. Cyclic voltammetry was run from −0.5 to 0.6 V at 50 mV s^–1^ and with an increasing number of scans (5, 10, and 30). Figure 3a depicts the cyclic voltammograms of the PB-MNs in PBS solution. As observed, all the signals presented an oxidation peak at ca. 150 mV and a reduction peak at ca. 0 mV. Previous observations reported in the literature related these peaks to the PW oxidation to PB and PB reduction to PW, respectively.^26^

The redox behavior of the PB-MN manifested in higher peak currents when the PB was prepared via the electrodeposition method (no matter what the number of scans) as opposed to the drop-casting chemical reaction approach (labeled as chemical in Figure 3a). Increasing the volume of the precursor solution used for the drop-casting resulted in much more mechanically unstable films; hence, we continued with the electrodeposition method rather than the drop-casting one. When the electrodeposition approach was applied with five cyclic voltammetric scans (from −0.5 to 0.6 V), the peak current and the reproducibility between subsequent cycles were slightly lower than with the 10- or 30-scan regimes. This behavior translated into a higher sensitivity for those MNs prepared with electrodeposited PB with 10 and 30 scans when the PB-MNs were tested regarding increasing H_2_O_2_ concentrations in amperometric mode (Figure S4). Considering the results in Figure 3a, an applied potential of −50 mV was selected for the reduction of PB to PW, thus generating the mechanism proposed in Figure 1d. No significant differences were found between the PB-MNs prepared with 10 and 30 scans; thus, the preparation with 10 scans was selected for further studies.

We then studied the effect of the amount of GLY-Ox deposited on the PB-based MN. Increasing volumes of the GLY-Ox/CHI mixture (2, 4, 6 and 8 μL) were deposited via drop-casting on top of the PB-MN. Figure 3b shows the amperometric responses to increasing GLY concentrations in the PBS background. As observed, a higher amperometric response, and therefore a higher slope, was displayed when the deposited volume of the enzyme solution was increased from 2 to 4 μL (sensitivities of −0.0449 nA μM^–1^ and −0.122 nA μM^–1^). This increase was not as drastic for higher volumes (slopes of −0.153 nA μM^–1^ and −0.130 nA μM^–1^ for 6 and 8 μL, respectively). In essence, the saturation of the enzyme is reached from a certain deposited volume/amount, because the available active sites will be the same. We decided to use a volume of 6 μL because it presented the maximum sensitivity and was enough to comfortably perform the drop-casting deposition in the modified MN.

Regarding the final external layer of Nafion, we confirmed that this was necessary to avoid any interference from ascorbic acid while preserving the response time and calibration graph of the sensor. This compound is of special concern because it can be directly oxidized in the PW/PB lattice, resulting in a less negative value for the current level provided by any GLY concentration.^27^ It was found that the addition of an external Nafion layer produced a slightly less noisy response at increasing GLY concentrations than a MN biosensor without that layer. Thus, the response time regarding increasing GLY concentrations was found to increase with the thickness of this layer (8 s versus 30 s), i.e., increasing the volume deposited on the MN, namely, 4 instead of 2 μL (Figure S5a). On the other hand, the ascorbic acid response was totally suppressed in the MN prepared with a volume of 2 μL, whereas without any Nafion layer, the MN biosensor responded to ascorbic acid (Figure S5b).

### Investigation of the Main Analytical Characteristics of the MN Biosensor for GLY Detection

The reversibility of the MN biosensor was demonstrated by measuring solutions with low and high GLY concentrations within the LRR in the following order: 50, 500, 50, 500, 50, and 500 μM (Figure S6). Almost negligible variations were found in the steady state currents (<2%), resulting in an averaged calibration graph with a sensitivity of −0.107 ± 0.001 nA μM^–1^ and an intercept of −76.7 ± 2.9 nA. The medium-term drifts observed in GLY concentrations of 50 and 500 μM were acceptably low (0.06 and 0.30 nA h^–1^, Figure S7), permitting the detection of these GLY concentrations with errors of <1% over 1 h. In addition, long-term drift experiments revealed variations of 0.12 and 1.28 nA h^–1^ for a 12-h period, and 0.23 and 1.38 for a 24-h period, for 50 and 500 μM (Figure S7). The lifetime of the MN biosensor was tested with daily calibrations: a decrease of 4% and ca. 40% of the sensitivity was found in the days 4th and 10th, while random changes appeared for the intercept. In view of these results, the MN biosensor is suitable to follow random increases and decreases in GLY concentration, with a rather reproducible response that is acceptably stable (and thus precise) over 12 h. For a longer continuous monitoring of GLY in ISF, it is advisable to either re-condition and re-calibrate the sensor or substitute it by a new patch. The MNs can be used after ca. 5 days of being prepared.

Glucose, lactate, pyruvate, urea, and ascorbic acid were investigated as potential interferences. The effects of concentrations higher than those expected in the ISF on the amperometric response were examined (Figure S8). It was confirmed that the biosensor qualifies as interference-free in the detection of GLY because none of the tested compounds influenced the GLY response. Notably, major ions, metabolites, and AAs commonly present in biological fluids, together with GLY derivatives, are not expected to influence the amperometric response, as previously demonstrated with a similarly structured biosensor and traditional specificity studies on the GLY-Ox enzyme.^17,28^

Calibration graphs were obtained at different pH in the background solution, while keeping the temperature (*T*) constant and vice versa, to study the influences of the pH and *T* on the response of the MN biosensor (Figure S9). These two factors may significantly alter the activity of the GLY-Ox enzyme. Under healthy conditions, the ISF pH and *T* are known to be independent of ambient fluctuations and subjects. However, the pH could slightly decrease and the *T* decrease/increase in patients with certain diseases.^29,30^ Accordingly, we selected a pH range from 6.5 to 7.5, and a *T* range from 25 to 40 °C in this study. It was observed that the dynamic responses, and consequently the calibration graphs, were almost identical regardless of pH and *T* values (see Table S1). The main differences appeared at the highest GLY concentration assayed (500 μM), although the most marked variations occurred because two different MN biosensors were used for the pH and *T* studies (2.9% of variation in the slope and ca. 30% in the intercept across all results). Hence, the developed MN biosensor can be calibrated at room temperature and at a pH of 7.5 for any posterior skin test.

**Figure 4:**
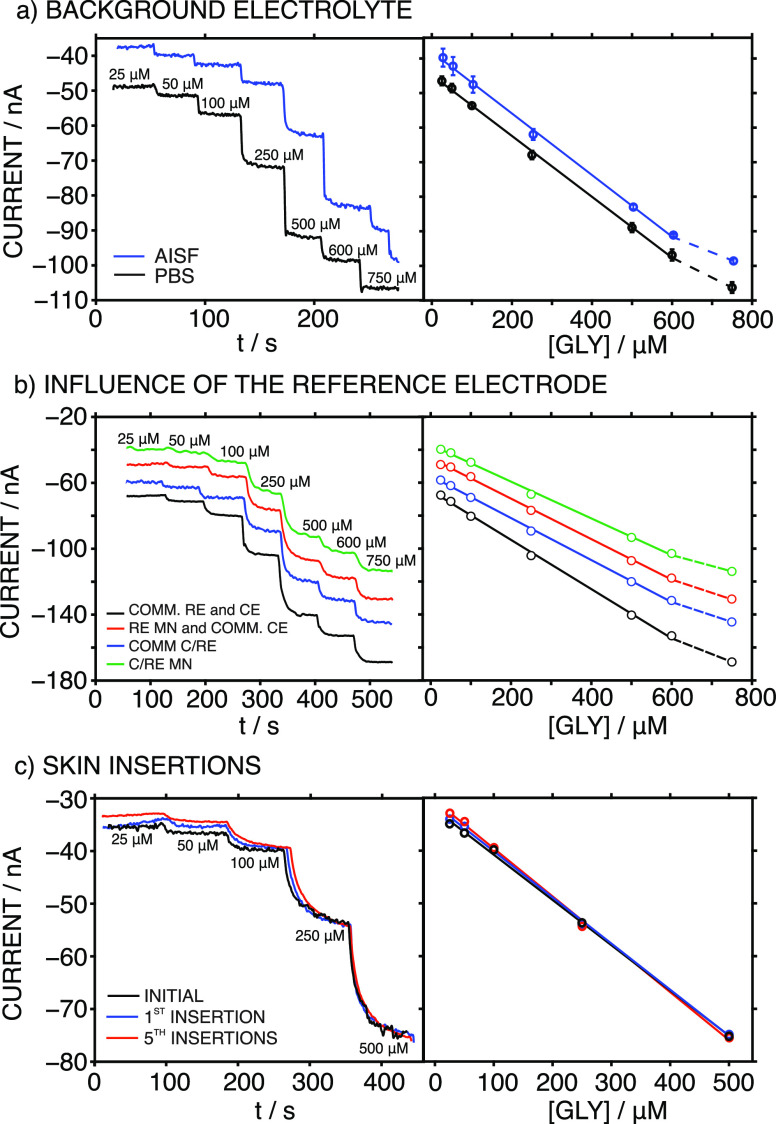
Dynamic amperometric responses and the corresponding calibration graphs for the MN biosensor at increasing GLY concentrations under different conditions: (a) PBS and AISF as the background electrolytes (the calibration graphs are the average of three sequential calibrations); (b) using commercial RE and CE, commercial C/RE, RE MN, and commercial CE and C/RE MN; (c) before and after insertions into rat skin.

Figure 4a depicts the dynamic responses at increasing GLY concentrations accomplished in either PBS (pH = 7.4) or artificial ISF (AISF, pH = 7.4) using the same MN biosensor. As observed, similar responses were obtained; however, there was a slight shift in the provided current (i.e., off-set). Thus, the calibration parameters were very similar, except for the intercept, representing excellent repeatability (three subsequent calibrations) independent of the background medium used for the calibration: LRRs for GLY concentrations of 25 to 600 μM; sensitivities (slopes) of −0.0880 ± 0.001 nA μM^–1^ (1.5% variation) and −0.0780 ± 0.004 nA μM^–1^ (5.3% variation) for PBS and AISF, respectively; and intercepts of −51.2 ± 1.9 nA (3.6% variation) and −36.0 ± 4.1 nA (11.4% variation) for PBS and AISF, respectively. Due to the observed changes in the intercepts of the linear graphs, it was most convenient to perform the calibration in the AISF medium, as this best mimics the real ISF where on-body measurements will be performed with the MN biosensor, thus minimizing any possible matrix effect.

Next, we investigated the possibility of using the MN GLY biosensor in conjunction with a C/RE MN (based on an Ag/AgCl element) to avoid implementing a three-electrode system in the final patch design, thus reducing the complexity and number of MNs necessary. Accordingly, the C/RE MN was expected to mediate the current flow through the WE while maintaining a constant potential.^31^ Notably, the current passing through the C/RE was relatively low (always lower than 0.1 μA); therefore, the risk of current induced changes in the Ag/AgCl-based MN leading to a change in the provided potential is expected to be negligible. Figure 4b displays the dynamic responses of the MN biosensor at increasing GLY concentrations in the AISF, together with the corresponding calibration graphs, when using different RE and CE systems: (i) commercial Ag/AgCl RE, and commercial Pt CE (COMM-RE); (ii) commercial Ag/AgCl RE acting as a C/RE; (iii) Ag/AgCl MN as the RE and commercial Pt as the CE; and (iv) the Ag/AgCl MN as the C/RE. It was evident that the LRR and slope were maintained independent of the CE and RE used (Table S2): the variations in the slope were always within the range displayed in the repeatability and reproducibility studies (ca. 13%). On the other hand, a higher variation was found for the intercept because of the different RE and CE pairs and different MN GLY biosensors used in the systems (23.3%). As a result, a two-electrode system composed of the MN GLY biosensor and the C/RE MN can be used without affecting the results. Therefore, this was the configuration used.

**Figure 5:**
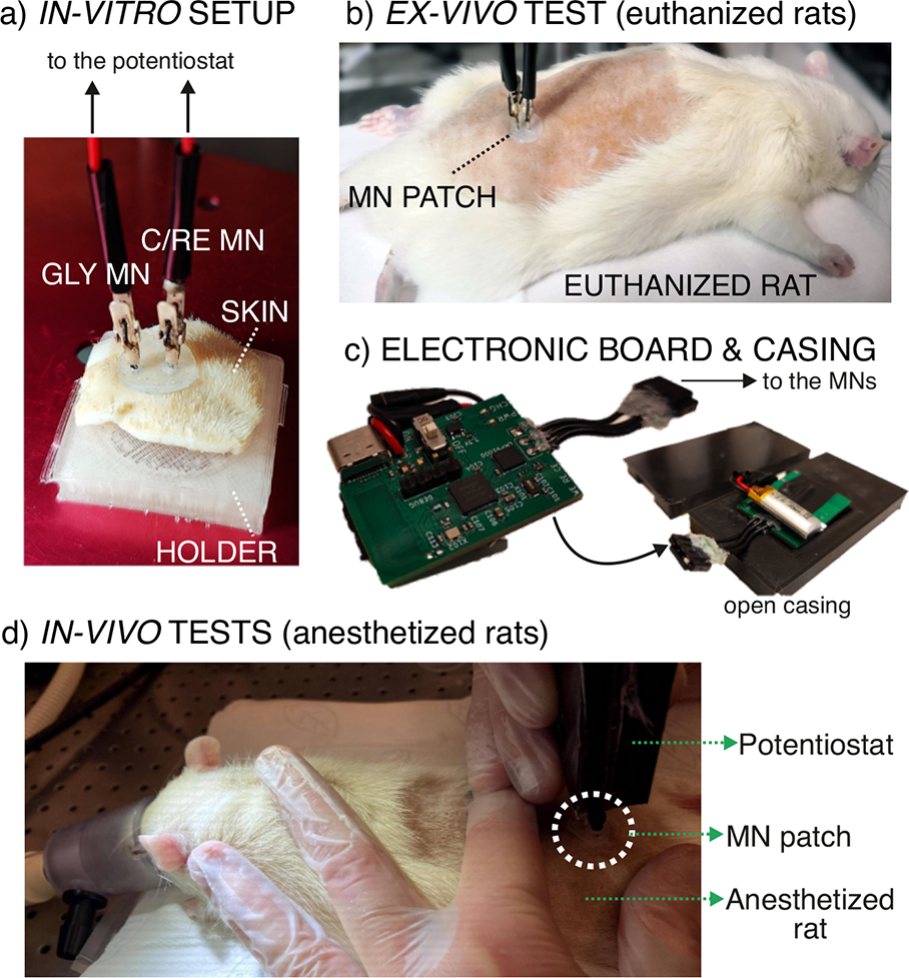
(a) Image of the in vitro setup, where a piece of rat skin is fixed in a holder to be analyzed with the MN biosensor. (b) Image of an on-body test using the MN GLY biosensor on a euthanized rat. (c) Image of the electronic board used for amperometric measurements, plus the casing used to introduce it and run the wireless data acquisition (KERIC). (d) Image of an in vivo test using the MN GLY biosensor in an anesthetized rat (UUBF).

Finally, it was confirmed that changes in the amperometric response of the two-electrode MN system were not induced by skin insertion. Moreover, the external modification made to the MNs to introduce the sensing elements did not detach and/or deteriorate. Calibration graphs in the AISF background were produced before and after one and five insertions in a piece of rat skin (Figure 4c), which was fixed in a holder to mimic on-body conditions (see Figure 5a for the in vitro setup). The results indicated that the calibration graph remained nearly invariable, with an LRR from 25 to 500 μM, an average slope of −0.0875 ± 0.002 nA μM^–1^ (2.7% variation), and an intercept of −31.0 ± 1.0 nA (3.1% variation). Effectively, the low variations indicated that the MN GLY biosensor can be initially calibrated and then used for successive skin insertions for the intradermal detection of GLY in the ISF without loss of integrity or biofouling. Moreover, SEM images of the MN biosensor and the C/RE MN revealed no significant changes or particle adherence on the MN’s surface (see Figure S1). Nevertheless, postcalibration graphs should be generated after skin insertions (in in vitro, ex vivo, and in vivo measurements) to confirm that the MN biosensor’s performance has not deteriorated.

### In Vitro Tests in Pieces of Rat Skin

Three pieces of rat skin (approx. 2 × 2 cm each) were conditioned for 24 h at 4 °C in the fridge in solutions containing GLY concentrations of 200, 300, or 400 μM in the PBS. Thereafter, each piece of skin was fixed in a 3D-printed holder (Figure 5a), allowed to dry at room temperature and squeezed with paper tissue to remove the remaining moisture. A patch containing the MN biosensor and the C/RE MN was implemented into the skin by carefully inserting the MNs, and the dynamic amperometric signal was recorded. For each skin, measurements were conducted through two insertions in different parts of the skin piece.

**Figure 6:**
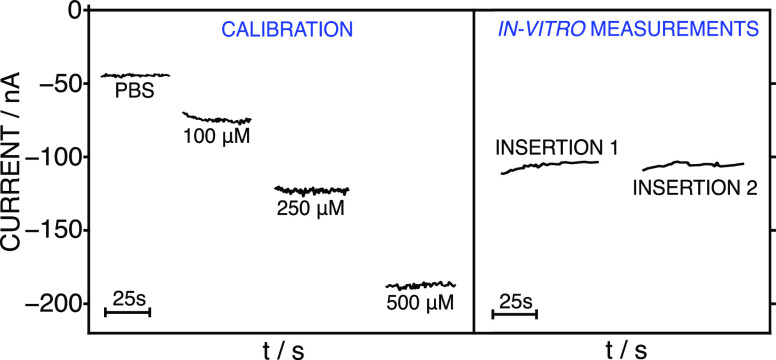
*Left:* Responses of the MN GLY biosensor at increasing GLY concentrations, which were used to obtain a calibration graph prior to the in vitro experiments. *Right:* Dynamic current registered during two consecutive insertions with the same MN patch (GLY biosensor and the C/RE MN) in a piece of rat skin conditioned in a 400 μM GLY solution.

Figure 6 exemplifies the amperometric response for a 3-point calibration graph accomplished previously to the in vitro experiment, together with the results of the two insertions in the skin conditioned in 400 μM GLY. The calibration was run in PBS background, the same as in the solution used for the skin conditioning. As observed, the recorded current in each insertion was fairly constant and the value was within the calibration graph. Thus, the calibration graph was employed to calculate the GLY concentration in each piece of skin and the results are collected in Table 1 as the average of two insertions.

**Table 1:** GLY Levels Detected in Different Samples and Rats by Means of Both the Developed MN GLY Biosensor and the Fluorescence Kit

sample/specimen	[GLY] average ± SD (μM)		% diff.
	MN^d^	fluorescence	
rat skin 1 (200 μM)^a^	72.5 ± 4.3	88.6 ± 0.6	18
rat skin 2 (300 μM)^a^	142.5 ± 3.5	111.6 ± 1.4	27
rat skin 3 (400 μM)^a^	216.7 ± 13.8	196.3 ± 11.9	10
rat 1 (euthanized)^b^	189.1 ± 7.4	193.6^e^	2
rat 2 (euthanized)^b^	103.0 ± 4.7	111.1^e^	7
rat 3 (euthanized)^b^	205.2 ± 25.7	202.1 ± 17.5	2
rat 4 (anesthetized)^c^	290.1 ± 14.4	232.6 ± 7.2	24

a In vitro experiments with rat skins conditioned in different GLY concentrations.

b On-body tests with euthanized rats at KERIC.

c In vivo tests with anesthetized rats at UUBF.

d The measurements averaged 4 to 6, as some outliers were identified.

e Only one measurement was possible due to insufficient volumes of collected ISF.

In addition, the ISF inside the skin was collected by a homemade peristaltic pump with a hollow MN hub (more details in the Supporting Information). In all cases, a volume of 1–3 μL was obtained. GLY contents in the collected ISF samples were analyzed with the commercial fluorescence kit for GLY detection in biological samples, and the results are provided in Table 1, along with the estimated results obtained with the MNs. Similar values were provided with both techniques, indicating the accuracy of the results obtained with the MN GLY biosensor. Notably, in all cases, the detected GLY concentration was lower than that in the corresponding conditioning solution. This behavior was previously noted in skin pieces conditioned in different ions (e.g., potassium) and biomolecules (such as urea) and attributed to specific partition and diffusion conditions.^19,32^

### Ex Vivo On-Body Measurements in Euthanized Rats (KERIC Facilities)

**Figure 7:**
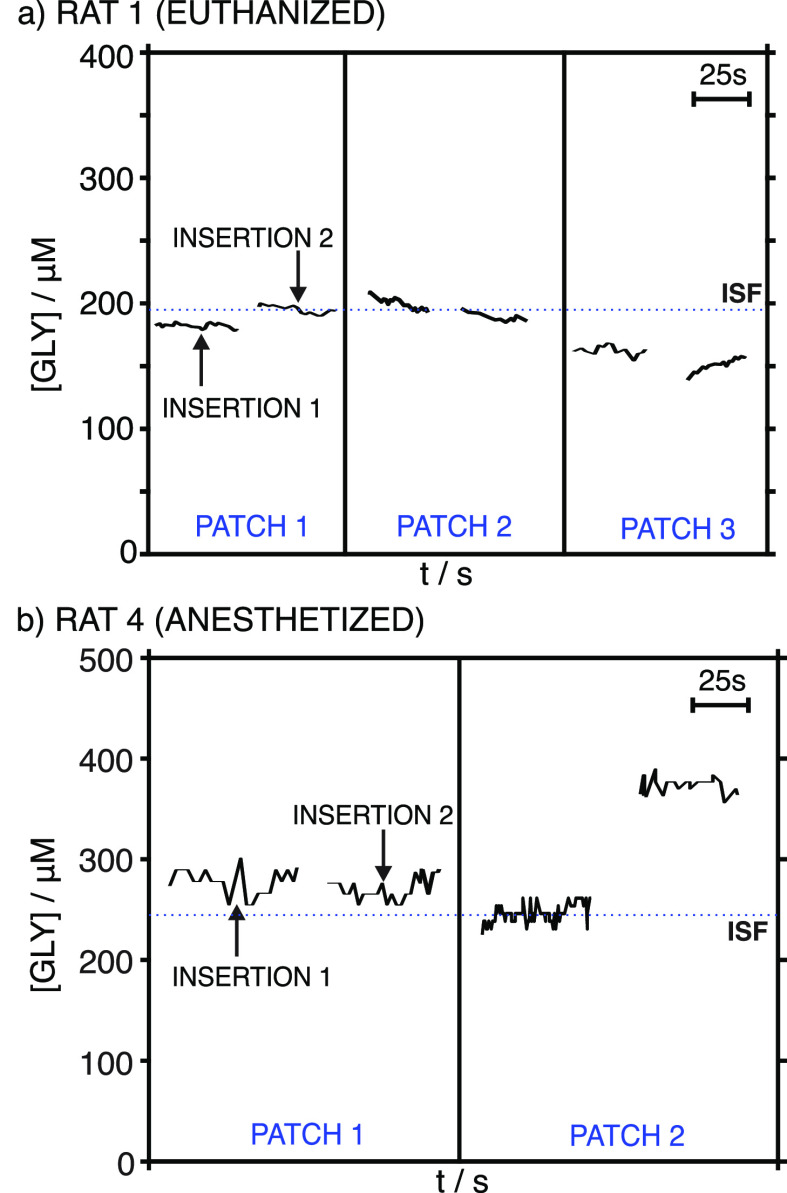
(a) Dynamic current registered in the ex vivo on-body test of (euthanized) rat 1 using three different MN patches, each one inserted twice in the shaved back of the rat. (b) GLY concentration registered during the in-vivo on-body test of (anesthetized) rat 4 using two different MN patches, each one inserted twice in the shaved back of the rat. Dashed lines indicate the levels of GLY found in collected ISF with the fluorescence assay kit.

The potential of the MN patch (MN biosensor and C/RE MN) to provide intradermal measurements of GLY was investigated in three euthanized rats. Figure 5b displays an image of one of the rats undergoing MN measurements on its shaved back region. Three different MN patches were inserted twice in different (but proximal) positions of the shaved back to collect current readings and transform them into GLY concentrations according to the calibration previously performed in AISF. Figure 7a presents all the GLY measurements for rat 1 as an example. As observed, similar measurements were obtained for the two insertions with each patch (variations of <5.5%) but also between the three MN patches (variations of <10%). For each rat, the GLY concentration was calculated as the average of four to six measurements, depending on the identification of any outliers due to malfunctions of the patch and/or electrical connections. The results are shown in Table 1.

In addition, ISF samples were collected from each rat, and the GLY content was analyzed by means of the fluorescence assay kit (Table 1). Interestingly, we found that the amount of ISF extracted (and collected) was related to age. Seemingly, younger rats have more ISF; thus, we were able to obtain 20–30 μL from 2-month-old rats, likely due to both their smoother skin and higher hydration levels compared to older rats.^33^ On the contrary, we could only obtain a few microliters from older rats, which sometimes made it difficult to validate the on-body measurements. Inspecting the estimated GLY levels, the MN and fluorescence methods produced very similar results, with differences of <7% between them, pointing out once more the accuracy of the MN measurements (Table 1).

### In Vivo On-Body Measurements of Anesthetized Rats (UUBF Facilities)

The developed MN patch was additionally used for intradermal measurements of GLY in three anesthetized rats (in accordance with the Uppsala Committee on Ethics of Animal, Dnr 5.8.18-18873/2018, DOUU-2020-025). In this case, the MNs (GLY biosensor and C/RE) were integrated into a miniaturized wireless potentiostatic board (Figure 5c). The board was placed inside a casing for protection and to facilitate portability during the in vivo on-body measurements (Figure 5c). Figure 5d shows one of the rats connected to the isoflurane flow (for anesthesia, details in the Supporting Information) while the on-body GLY measurements with the MN patch were performed. Two different MN patches were tested on the rats via two insertions in different (but proximal) positions on their shaved backs. Unfortunately, conclusive measurements were only possible for one of the rats (labeled as rat 4), with the absence of reproducible data for the other two due mainly to problems with the MN connections to the electronic board. Figure 7b displays all the GLY measurements acquired from rat 4. While very similar data were obtained in the two insertions of the first patch and the first insertion of the second patch, the last measurement rather deviated from the rest and was not considered in the final calculation of the GLY concentration. The results are also found in Table 1. As observed, a higher difference between the MN readings and fluorescence kit results was found for the ISF GLY (>20%), compared to the case for euthanized rats. The results chiefly demonstrated the possibility that rather accurate intradermal measurements of GLY can be made.

## Conclusions

The very first MN biosensor for transdermal GLY analysis in ISF has been herein presented. The solid stainless steel MNs have been externally tailored with a series of elements for the specific and selective determination of GLY. The performance of the MN GLY biosensor has been deeply investigated, and it has been found to have a fast response time; good reversibility and reproducibility; a negligible response to pH, *T*, and the main interfering agents present in ISF; and an LRR suitable for GLY analysis in ISF. Validated in vitro assays using pieces of rat skin, together with ex vivo and in vivo on-body tests in rats, have successfully demonstrated the capability of the developed MN biosensor in terms of providing accurate intradermal GLY measurements in dermal ISF. Remarkably, to the best of our knowledge, these are the very first on-body transdermal measurements in ISF that are reported in rats. The developed experimental path may serve as a practical guide for the appropriate analytical characterization of any newly developed MN (bio)sensor. Furthermore, the results herein constitute meaningful advances in the development and application of wearable eHealth devices based on minimally invasive MNs. For GLY detection, it is expected that the developed device contributes to generate unprecedented data concerning the connection of GLY levels in ISF with its use as a biomarker and/or in disorders’ treatments.

## Supporting Information Available

Experimental details; procedures for on-body tests; SEM images; reproducibility; electrochemical behavior of the PB-MN; optimization of the external Nafion layer; reversibility; drift; interferences; pH and T influences (PDF)
