# Supplementary material for: Intradermal Glycine Detection with a Wearable Microneedle Biosensor: The First In Vivo Assay
Source: Anal Chem. 2022 Aug 18;94(34):11856–64. doi: 10.1021/acs.analchem.2c02317 (PMC9434558; doi:10.1021/acs.analchem.2c02317)
Supplement: Supplementary file 1 — ac2c02317_si_001.pdf [file ac2c02317_si_001.pdf]

Supporting information for:

Intradermal Glycine Detection with a Wearable Microneedle Biosensor: The First In-Vivo Assay

Qianyu Wang[1], Águeda Molinero-Fernández[1], Ana Casanova[1], Joep Titulaer[2], Jonatan C. Campillo-Brocal[3], Åsa Konradsson-Geuken[2], Gastón A. Crespo[1] and Maria Cuartero[1,*]

[1] Department of Chemistry, School of Engineering Sciences in Chemistry, Biotechnology and Health, KTH Royal Institute of Technology, Teknikringen 30, SE-100 44, Stockholm, Sweden.
[2] Section of Neuropharmacology and Addiction Research, Department of Pharmaceutical Biosciences, Uppsala University, Uppsala, Sweden.
[3] Department of Genetics and Microbiology, University of Murcia, Campus Universitario de Espinardo, Murcia, Spain.

(*) Corresponding Author: Maria Cuartero (mariacb@kth.se)

Table of Contents

1. Experimental Section ... *3*

1.1.Reagents, materials and instrumentation. ... 3

1.2.Preparation of GlyOx.3 ... 3

1.3.Fabrication of the substrate for the MN patch. ... 4

1.4.Ex-vivo experiments in euthanized rats. ... 4

1.5.In-vivo experiments in anesthetizied rats. ... 5

1.6.Validation of intradermal measurements. ... 5

2. Tables ... *6*

Table S1. ... 6

Table S2. ... 6

3. Figures ... *7*

Figure S1. ... 7

Figure S2. ... 7

Figure S3. ... 8

Figure S4. ... 8

Figure S5. ... 9

Figure S6. ... 9

Figure S7. ... 10

Figure S8. ... 10

Figure S9. ... 11

4. References ... *12*

1. Experimental Section

1.1. Reagents, materials and instrumentation. Sodium chloride (CAS-7647-14-5), potassium chloride (CAS-7447-40-7), sodium phosphate dibasic (CAS-10028-24-7), potassium phosphate monobasic (CAS-7778-77-0), glycine (CAS-56-40-6), acetic acid (CAS-64-19-7), Nafion® perfluorinated resin solution 5% (CAS- 31175-20-9), polyurethane (CAS-51-79-6), polyvinyl butyral (CAS-63148-65-2), Iron (III) chloride (CAS-7705-08-0), potassium ferricyanide (CAS-13746-66-2), calcium chloride (CAS-22691-02-7), tetrahydrofuran (CAS-109-99-9), sodium bicarbonate (CAS-144-55-8), magnesium chloride (CAS-7786-30-3), L-ascorbic acid (CAS-50-81-7), hydrogen peroxide (CAS-7722-84-1), bovine serum albumin (CAS- 9048-46-8) were purchased from Sigma Aldrich. Hydrochloric acid (CAS-7647-01-0), sodium pyruvate (CAS-113-24-6), uric acid (CAS-69-93-2), creatine monohydrate (CAS-6020-87-7), creatinine (CAS-60-27-5), D-glucose (CAS-50-99-7), sodium L-lactate (CAS-867-56-1), sodium dihydrogen citrate (CAS-6132-04-3), and urea (CAS-57-13-6) were purchased from Alfa Aesar. Sodium hydroxide (CAS-1310-73-2), ethanol absolute (CAS-64-17-5), and glycerol (CAS-56-81-5), were purchased from VWR. Chitosan (CAS-9012-76-4) was purchased from ACROS ORGANICS. Silver/silver chloride (Ag/AgCl) ink (C2131007D3) and carbon ink (C2030519P4) were obtained from Gwent group, UK.

All solutions were prepared in 18.2 MΩ cm^{-1} doubly deionized water (Milli-Q water systems, Merck Millipore). Phosphate Buffer was prepared by mixing KH_2PO_4 and Na_2HPO_4 to reach a concentration of 0.01 M, with the pH value set at 7.4. Phosphate buffered saline solution (PBS) was prepared by mixing NaCl, KCl, Na_2HPO_4, and KH_2PO_4 to reach the concentration of 0.01 M, with the pH value set at 7.4. Artificial interstitial fluid (a-ISF) was prepared as described elsewhere and without containing glycine.[1,2] Briefly, the composition of a-ISF was 20 g L^{-1} BSA, 3.5 mM KCl, 1.5 mM $CaCl_2$, 0.7 mM $MgCl_2$, 140 mM NaCl, 26 mM $NaHCO_3$, 1.7 mM Na_2HPO_4, 6 mM glucose and 7 mM urea.

The microscopic images were obtained by a scanning electron microscope (FE-SEM Hitachi S-4800, Japan). Amperometric measurements were performed with a potentiostat (Autolab, Metrohm Nordic AB, Sweden) against a single junction Ag/AgCl/sat.KCl reference electrode (Metrohm Nordic Sweden) or the MN-based reference or counter/reference electrode. A hand-made amperometric board with wireless data transmission was used for the rat-based experiments.

For the *in-vitro* experiments, rat skins of euthanized specimens were cut into squared pieces and stored in the freezer at –18 °C. Before being used, each piece of skin was thoroughly cleaned with distilled water and being then conditioned in the selected glycine concentration / PBS solution and stored in the fridge at 2 °C for 24 hours prior to the assay, which saw performed by means of a special support to fix the skin (produced by 3D printing). Ex-vivo experiments involving donated euthanized rats were performed at the Karolinska University Hospital (Sweden) assisted by the operation manager and Karolinska Experimental Research and Imaging Centre (KERIC) personnel, using specimens that were euthanized for other researcher purposes rather than those in this paper. In-vivo experiments with anesthetized rats were carried out at the Behavior Facility in Uppsala University (UUBF).

1.2. Preparation of GlyOx.[3] For recombinant expression of GlyOx, one BL21(DE3) Escherichia coli colony, containing the pETGoxAB15 plasmid,[4] was incubated overnight in 5 mL of lysogeny broth (LB) at 37 °C and 200 rpm. The overnight culture was used to inoculate 400 mL of terrific broth (TB) medium plus 1% glucose, which was further incubated at 200 rpm

and 37 °C. At OD600 0.7–0.9, 1 mM of IPTG was added to the culture and the temperature was decreased to 15 °C. Both media were supplemented with 50 µg/ml ampicillin. After 16 h of induction, cells were harvested by centrifugation (Beckman coulter Avanti J-26XP) at 11 000 g at 4 °C for 30 min. The collected pellet was resuspended in binding buffer for 6 min disruption by sonication at 30 duty cycles % and with a 5–6 Microtip limit in a Branson Sonifier 250. Then, after 30 min of centrifugation at 4 °C and 3500 g, the pellet was discarded, and the supernatant was collected and filtered (0.45 µm). Purification was performed on a Ni-NTA His-trap column, according to the IBA manufacturer′s manual with binding buffer (50 mM NaH_2PO_4, 500 mM NaCl, 20 mM imidazole, pH 7.4) and elution buffer (50 mM NaH_2PO_4, 500 mM NaCl, 500 mM imidazole, pH 7.4). After purification, samples were buffer exchanged with PD-10 desalting columns (GE Healthcare) to a buffer containing 50 mM NaH_2PO_4, 500 mM NaCl, pH 7.4. Protein concentrations of pure GlyOx samples were measured on a Nanodrop (Thermo Fisher) using the E 0.1% values calculated in ProtParam (ExPASy server).

The ABTS peroxidase colorimetric assay was used to determine the GlyOx activity in pure GlyOx samples. This assay is based on the oxidation of ABTS (2,2'-Azino-bis(3-Ethylbenzthiazoline-6-Sulfonic Acid)) catalysed by horseradish peroxidase (HRP) in the presence of the hydrogen peroxide produced by GlyOx. The ABTS oxidation was followed by measurement of the absorption at 420 nm in a FLUOstar Optima (BMG LabTech). The assay mixture (100 µL) routinely contained 20 mM of glycine in 50 mM NaH2PO4, 500 mM NaCl, pH 7.4, with 10 mM ABTS, 0.1 U/mL of peroxidase, and 25 µg of pure GlyOx. Reactions were carried out at 37°C for 15 min in 96-well ELISA (enzyme-linked immunosorbent assay) plates. In order to obtain the enzyme activity in pure samples (U/mL or U/mg of GlyOx), a standard curve with hydrogen peroxide was assayed.

1.3. Fabrication of the substrate for the MN patch. The substrate was fabricated by mixing equal volumes of solutions labelled as ‘Part A’ and ‘Part B’ of the commercial pourable silicon rubber (Ecoflex™ 00-50, Smooth-on Inc.) and filling a 3D printed mould of 13 mm of diameter and 1 mm of depth with the resulting mixture. Afterwards, the substrate was allowed to cure for 3 h, according to the manufacturer instructions.

1.4. Animals in UUBF. Adult male Sprague-Dawley rats, 7–8 weeks of age (Envigo, Horst, The Netherlands), weighing approximately 180 grams at arrival were used for *in-vivo* experiments. All animals were housed under standard laboratory conditions with constant room temperature (~22°C) and relative humidity (~65%) in groups of 2–5 with access to food and water ad libitum and were maintained on a 12-hour light/dark cycle (lights on at 06.00 or, reversed light/dark cycle for rats used in the CAR experiment with lights off at 06.00). Animals arrived at least 5 days prior to any of the experiments. The experiments were approved by and conducted in accordance with the Uppsala Committee on Ethics of Animal (Dnr 5.8.18-18873/2018, DOUU-2020-025).

1.5. Ex-vivo experiments in euthanized rats. The experiment involved three rats in total. Bio-breeding diabetes-prone rats (BBDP-rats) euthanized in a CO_2 chamber (gas flow rate of 1/4 of the container per minute) for other research purposes rather than those in this paper, and donated by KERIC, were employed. In particular, the specimens weighed 270–280 g each, including both males and females. We shaved the rat's back fur and sterilized it with ethanol and then, previously calibrated MN patches were inserted through the skin. The current was recorded until a steady state value was reached for at least 1 min using the hand-made potentiostat board. Several insertions and in different back positions were accomplished. Thereafter, the rats’ backs were opened with a scalpel, exposing the subcutaneous tissue. ISF was extracted from the internal part of the skin by using a homemade device consisting of a plastic hub containing 4 hollow MNs (0.24×0.11×10 mm, Micropoint Techno logies Pte

Ltd, Singapore). The hub was in turn coupled to a a small PFTE tubing (Sigma-Aldrich, 0.3 and 0.6 mm of inner and outer diameter) connected to a bigger one (Tygon LMT-55, ISMATEC, Cole-Parmer GmbH, Germany) that allows us the assembling with a peristaltic pump (ISMATEC IPC series, Cole-Parmer GmbH, Germany). The ISF extraction was performed just after on-body MN sensing to minimize the possible alterations of the ISF either in quantity or quality, given the fact that the rat is no longer alive. In most of the cases, 30 min to extract a useful amount of ISF for further studies. Finally, rat skin samples were collected in the falcon.

1.6. *In-vivo* experiments in anesthetized rats. Three male Sprague Dawley rats from Envigo (the Netherlands) that were between 7-8 weeks old were used in the experiment. Initial anaesthesia was performed with 4% isoflurane, once the rats were mounted in the stereotactic frame (David Kopf Instruments, AgnTho's, Lidingö, Sweden) the isoflurane level was adjusted according to the breathing of the animal (isoflurane with air (2-4 L/min, 2-2.5% v/v). A heating pad was placed under the animal and artificial tears (Viscotears, Bausch Lomb Nordic, Stockholm, Sweden) were applied to the eyes of the rat. Afterwards, the back of the rat was shaved and sterilized with ethanol. The skin of the rat was carefully pierced with the MN patch and data was recorded for ~5 minutes, after which the MN patch was inserted at a different position and the measurements procedure was repeated. After the experiment, the rat was decapitated, and the skin of the back was removed, and a hollow MNs-device was used to collect ISF from inside the skin. Finally, rat skin samples were collected for analysis.

1.7. Validation of intradermal measurements. Validation measurements were accomplished by means of a commercially available fluorescence kit (Glycine Assay Kit (Fluorometric), Sigma Aldrich). The standard glycine solutions and the samples were added in a measuring plate (96 Well Black Plate, Nunclon™ Delta Surface, Thermo Fisher Scientific) according to the kit instructions. The samples were subsequently incubated at 25 °C for 1h (Termaks incubator type B 8133, Termaks AS, Norway) and later analyzed (MARS Data Analysis Software, BMG Labtech, Germany) in the microplate reader (CLARIOstar, BMG LABTECH, Germany). All the standards and samples were run at least in duplicate, depending on the available sample volume. The fluorescence intensity was measured at Ex/Em = 535/587 nm.

2. Tables

Table S1. Parameters and variation coefficients for the MN GLY biosensor at different pH and temperature conditions.

Conditions	Slope (nA/µM)	Intercept (nA)
25 °C, pH 6.5	–0.132	–65.8
25 °C, pH 7.0	–0.141	–53.8
25 °C, pH 7.5	–0.139	–43.3
pH 7.5, 37 °C	–0.133	–28.9
pH 7.5, 40 °C	–0.139	–36.6
Average	–0.137	–45.7
Standard Deviation	0.004	14.5
RSD	2.9 %	31.7 %

Table S2. Slope and intercept of the calibration graphs for the MN GLY biosensor using different RE and CE configurations.

	COMM. RE + COMM. CE + MN WE	COMM. C/RE + MN WE	COMM. CE + MN RE + MN WE	MN C/RE + MN WE
Slope (nA/µM)	–0.143	–0.122	–0.117	–0.106
Intercept (nA)	–65.6	–56.7	–46.3	–38.0

3. Figures

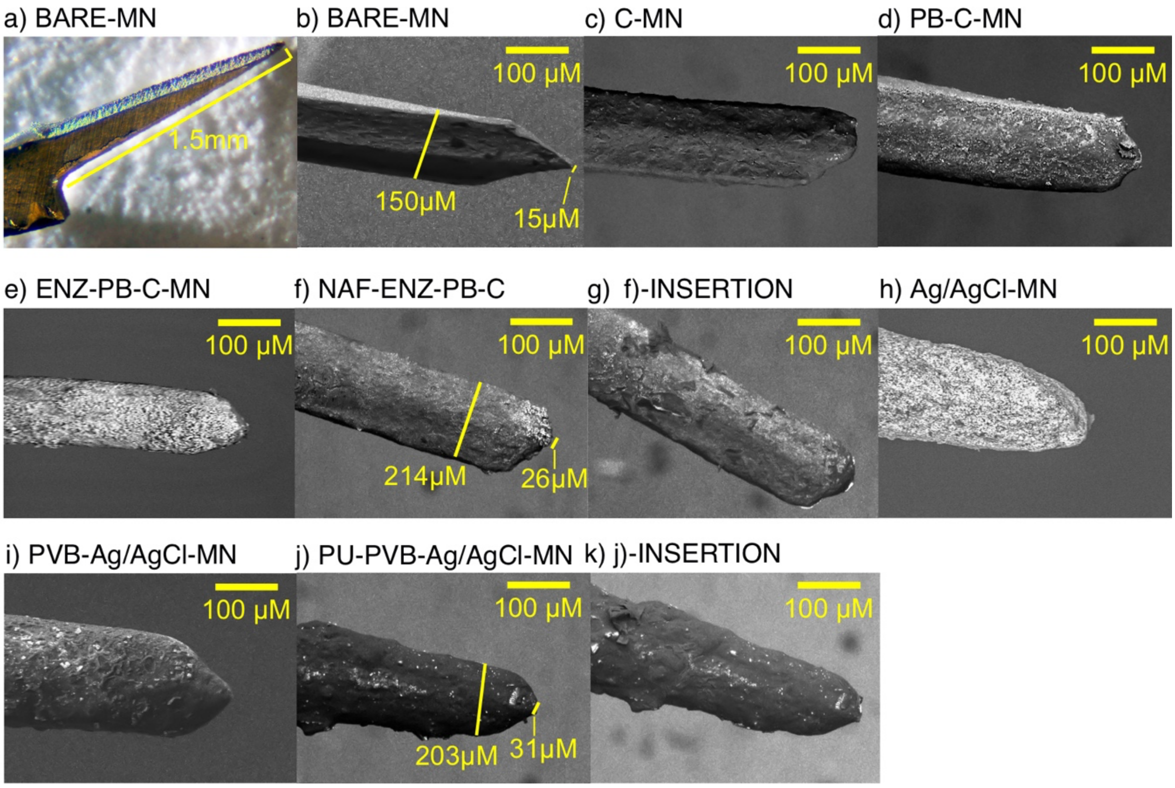

Figure S1. (a) Optical image of a bare MN. SEM images of (b) bare MN, (c) C-MN, (d) PB-C-MN, (e) GLY-Ox/CHI-PB-C-MN, (f) Nafion-GLY-Ox/CHI-PB-C-MN, (g) the same Nafion-GLY-Ox/CHI-PB-C-MN after skin insertion, (h) Ag/AgCl-MN, (i) PVB-Ag/AgCl-MN, (j) PU-PVB-Ag/AgCl-MN, and (k) the same PU-PVB- Ag/AgCl-MN after skin insertion.

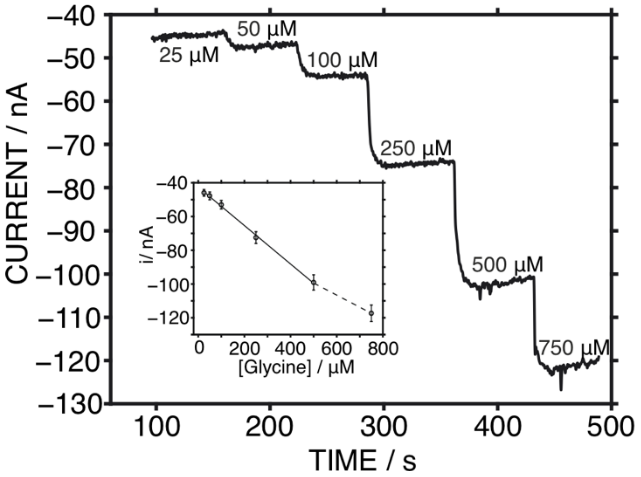

Figure S2. Dynamic amperometric response at increasing GLY concentrations. Applied potential of −0.05V versus the commercial Ag/AgCl reference electrode. Inset: average calibration graph observed for seven MN biosensors, including the one corresponding to the dynamic response.

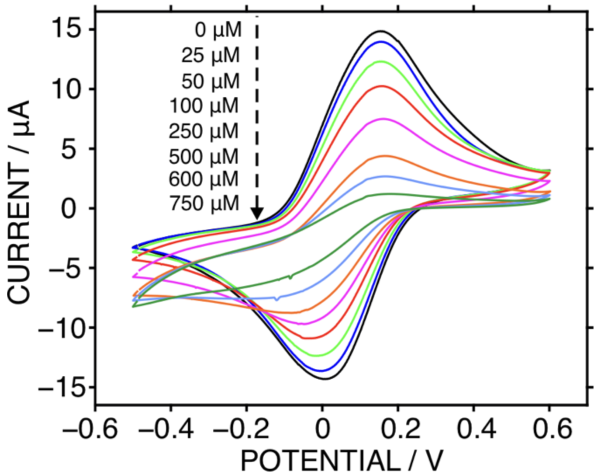

Figure S3. For a PB-C-MN electrode in PBS (pH = 7.4), cyclic voltammograms at increasing H_2O_2 concentrations. Scan rate = 50 mV s^{-1}.

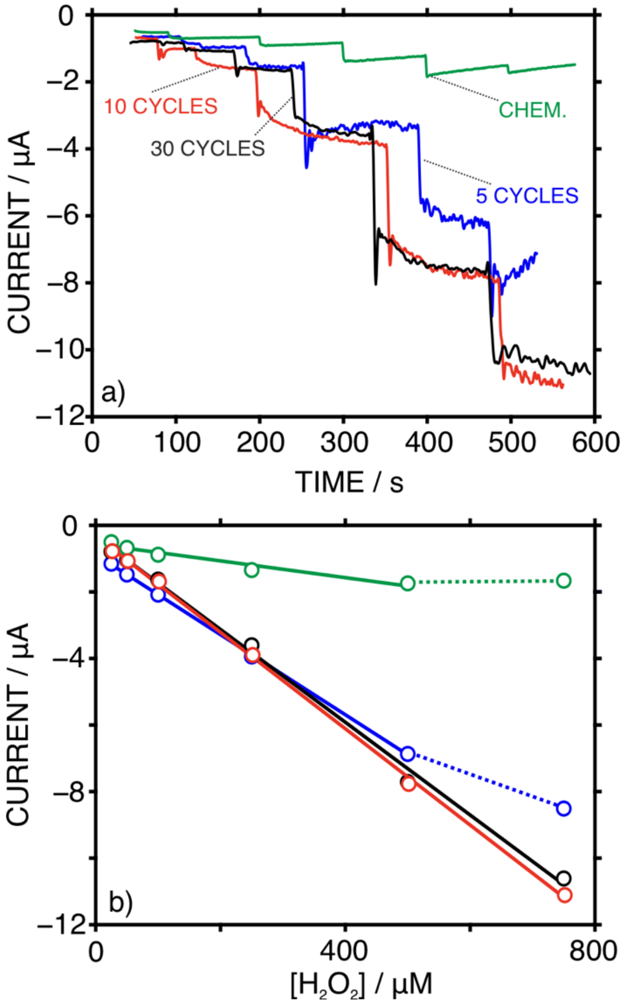

Figure S4. For a PB-C-MN electrode in PBS (pH = 7.4): (a) Amperometric response at increasing H_2O_2 concentrations. Applied potential of –0.05V versus the commercial Ag/AgCl reference electrode. PB preparation methods are presented with different colors. Green: chemical reaction method; blue: electrodeposition with 5 CV scans; red: electrodeposition with 10 CV scans; black: electrodeposition with 30 CV scans. (b) Corresponding calibration curves.

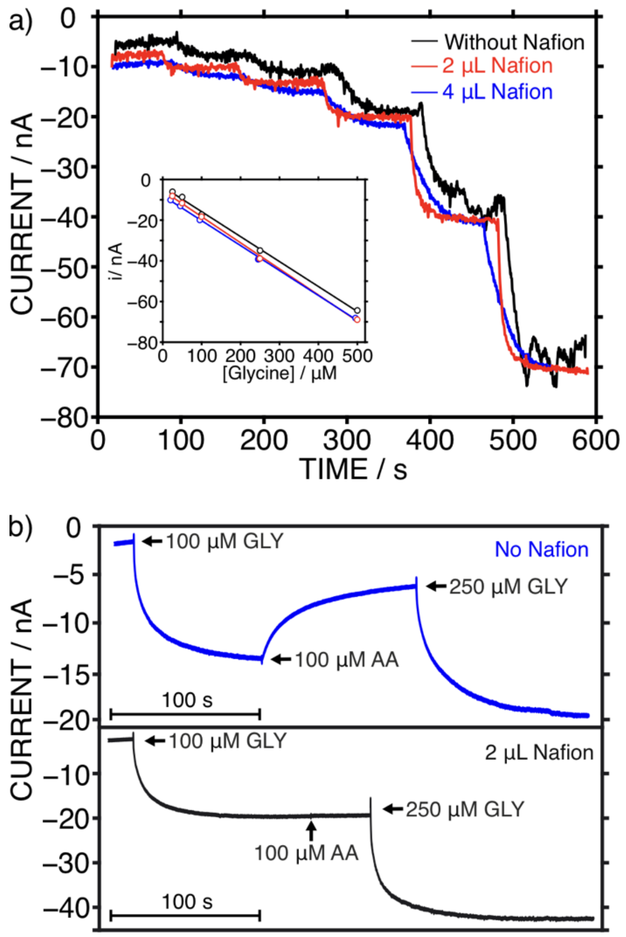

Figure S5. (a) Dynamic responses of GlyOx-PB-C-MN electrode prepared without or with 2 µL and 4 µL Nafion membrane. Inset: corresponding calibration curves. (b) Dynamic responses toward the addition of 100 µM GLY→100 µM ascorbic acid (AA)→250 µM GLY. Top figure: GlyOx-PB-C-MN electrode prepared without Nafion membrane; bottom figure: GlyOx-PB-C-MN electrode prepared with 2 µL Nafion membrane.

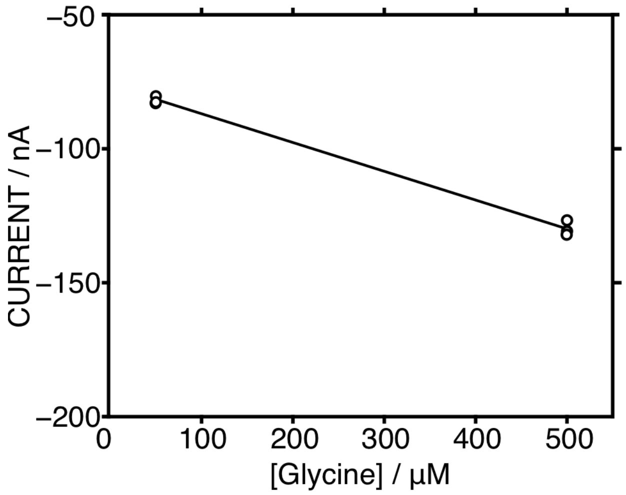

Figure S6. Average calibration graph obtained in the reversibility study. The biosensor response followed the sequence of 50→500→50→500→50→500 µM for the glycine concentration.

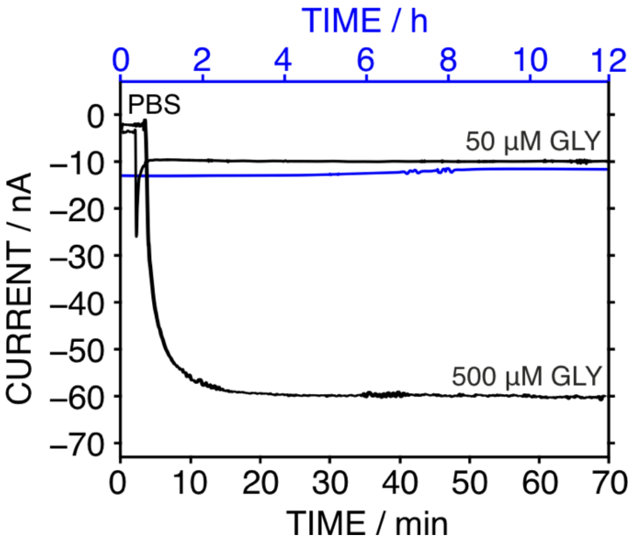

Figure S7. *Black lines:* Medium-term amperometric response of 50 and 500 µM glycine concentrations. The PBS background was modified with pertinent additions of glycine to reach 50 and 500 μM concentration and then, the current was registered over ca. 1 h. *Blue line:* Long-term amperometric response of 50 µM glycine concentration. The biosensor was directly immersed in a solution containing 50 µM glycine concentration in the PBS background.

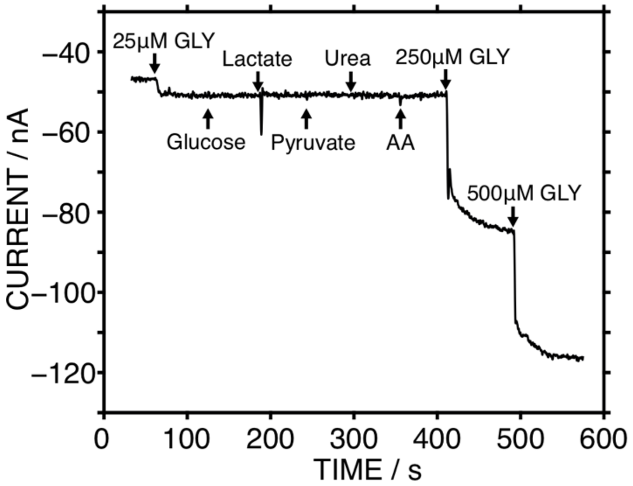

Figure S8. Dynamic response at increasing glycine concentrations after adding to the background solution (phosphate buffer at pH of 7.4) interferences: 6 mM glucose, 1.2 mM lactate, 120 µM pyruvate, 7 mM urea, 100 µM AA.

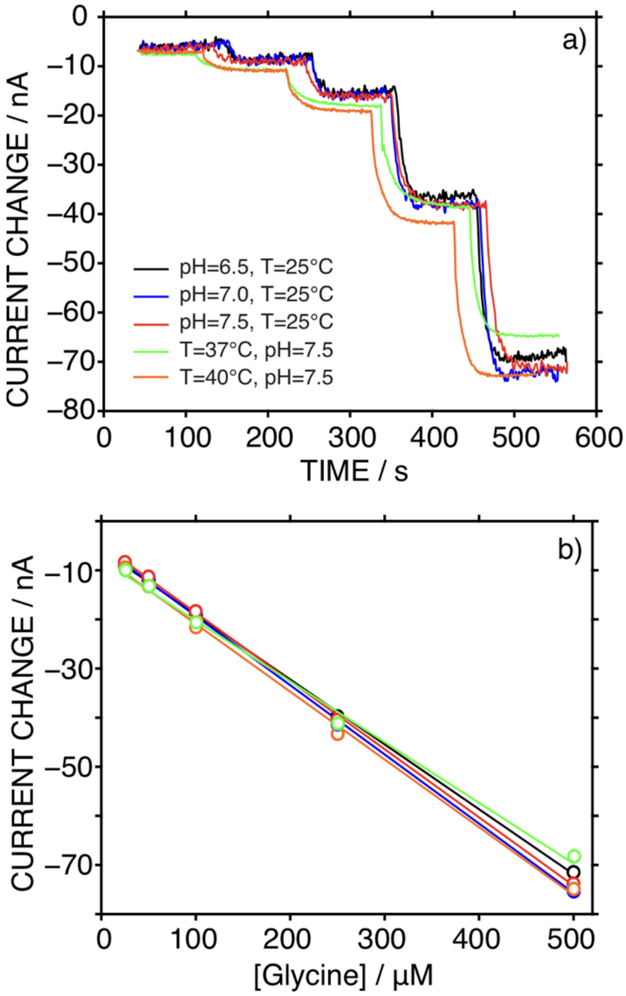

Figure S9. (a) Dynamic responses of GLY-MN biosensor at different pHs and temperatures. Black: pH = 6.5, T = 25 °C; blue: pH = 7.0, T = 25 °C; red: pH = 7.5, T = 25 °C; green: T = 37 °C, pH = 7.5; orange: T = 40°C, pH = 7.5. (b) Corresponding calibration curves.

4. References

(1) Bretag, A. H. Synthetic interstitial fluid for isolated mammalian tissue. *Life Sci.* **1969**, *8* (5), 319-329.

(2) Fogh-Andersen, N.; Altura, B. M.; Altura, B. T.; Siggaard-Andersen, O. Composition of interstitial fluid. *Clin. Chem.* **1995**, *41* (10), 1522-1525.

(3) Campillo-Brocal, J. C.; Lucas-Elio, P.; Sanchez-Amat, A. Identification in M arinomonas mediterranea of a novel quinoprotein with glycine oxidase activity. *Microbiologyopen* **2013**, *2* (4), 684-694.

(4) Chacón-Verdú, M. D.; Campillo-Brocal, J. C.; Lucas-Elío, P.; Davidson, V. L.; Sánchez-Amat, A. Characterization of recombinant biosynthetic precursors of the cysteine tryptophylquinone cofactors of l-lysine-epsilon-oxidase and glycine oxidase from Marinomonas mediterranea. *Biochim Biophys Acta Proteins Proteom* **2015**, *1854* (9), 1123-1131.
